# Primary Xp11 translocation PEComa of the testis with *SFPQ*⁃*TFE3* rearrangement: a case report and review of the literature

**DOI:** 10.1186/s13000-023-01288-x

**Published:** 2023-01-16

**Authors:** Huizhi Zhang, Suying Wang, Lingli Meng

**Affiliations:** Department of Pathology, Ningbo Clinical Pathology Diagnosis Center, Zhejiang Ningbo, China

**Keywords:** Xp11 translocation, Melanocytic differentiation, *TFE3*, Perivascular epithelioid cell tumor, Testis

## Abstract

**Background:**

Perivascular epithelioid cell neoplasms (PEComas) are a family of mesenchymal tumors with features of both smooth muscle and melanocytic differentiation. A subset of PEComas demonstrate rearrangements involving the *TFE3* (Xp11) locus. Xp11 translocation PEComa is a rare neoplasm with special clinicopathological features and a more aggressive behavior. We recently encountered a case of Xp11 translocation PEComa occurring in the testis, with *SFPQ*⁃*TFE3* rearrangement.

**Case presentation:**

A 57-year-old male touched a mass in his testis incidentally. MRI revealed a 10 mm diameter mass in the right testis. The patient underwent radical orchiectomy. Gross examination revealed a well-demarcated mass from the surrounding testicular tissue. Microscopically, the tumor mainly displayed nested or sheet-like architecture separated by delicate fibrovascular septa. The tumor cells exhibited marked nuclear atypia and pleomorphism. Immunohistochemistry showed that the tumor cells were strongly positive for cathepsin-K, HMB45 and TFE3. Molecular analysis revealed *SFPQ*⁃*TFE3* gene fusion. Thus, it was diagnosed as primary Xp11 translocation PEComa of the testis.

**Conclusions:**

The present case reports primary Xp11 translocation PEComa of the testis for the first time, which to our knowledge has not been described in the literature in this anatomic site, where it could potentially be problematic in diagnosis.

## Introduction

Perivascular epithelioid cell tumors (PEComas) are a group of rare mesenchymal neoplasms composed of perivascular epithelioid cells exhibiting both melanocytic and muscular differentiation. The majority of PEComas show genetic alterations in *TSC2* (the result of a loss of heterozygosity in the *TSC2* gene) and, less commonly, *TSC1* [1]. A small subset of PEComas harbour *TFE3* (Xp11) gene fusions, which show unique characteristics, as initially described by Argani et al. [2] in 2010 and considered to represent an unusual variant of PEComa. PEComas of the urinary system and male genital organs are extremely rare mesenchymal neoplasms that have been described mainly in the kidney but may also occur in other locations. Approximately 40 cases of PEComas of the urinary bladder and 4 cases of PEComas of the prostate have been reported in the literature; only 9 cases of bladder PEComas and 1 case of prostatic PEComas with *TFE3* rearrangement have been described in the literature [3–5]. We present a case of Xp11 translocation PEComa occurring in the testis harboring *SFPQ*⁃*TFE3* rearrangement in a 57-year-old male, which, to the best of our knowledge, represents the first case of primary Xp11 translocation PEComa of the testis.

## Case presentation

**Fig. 1:**
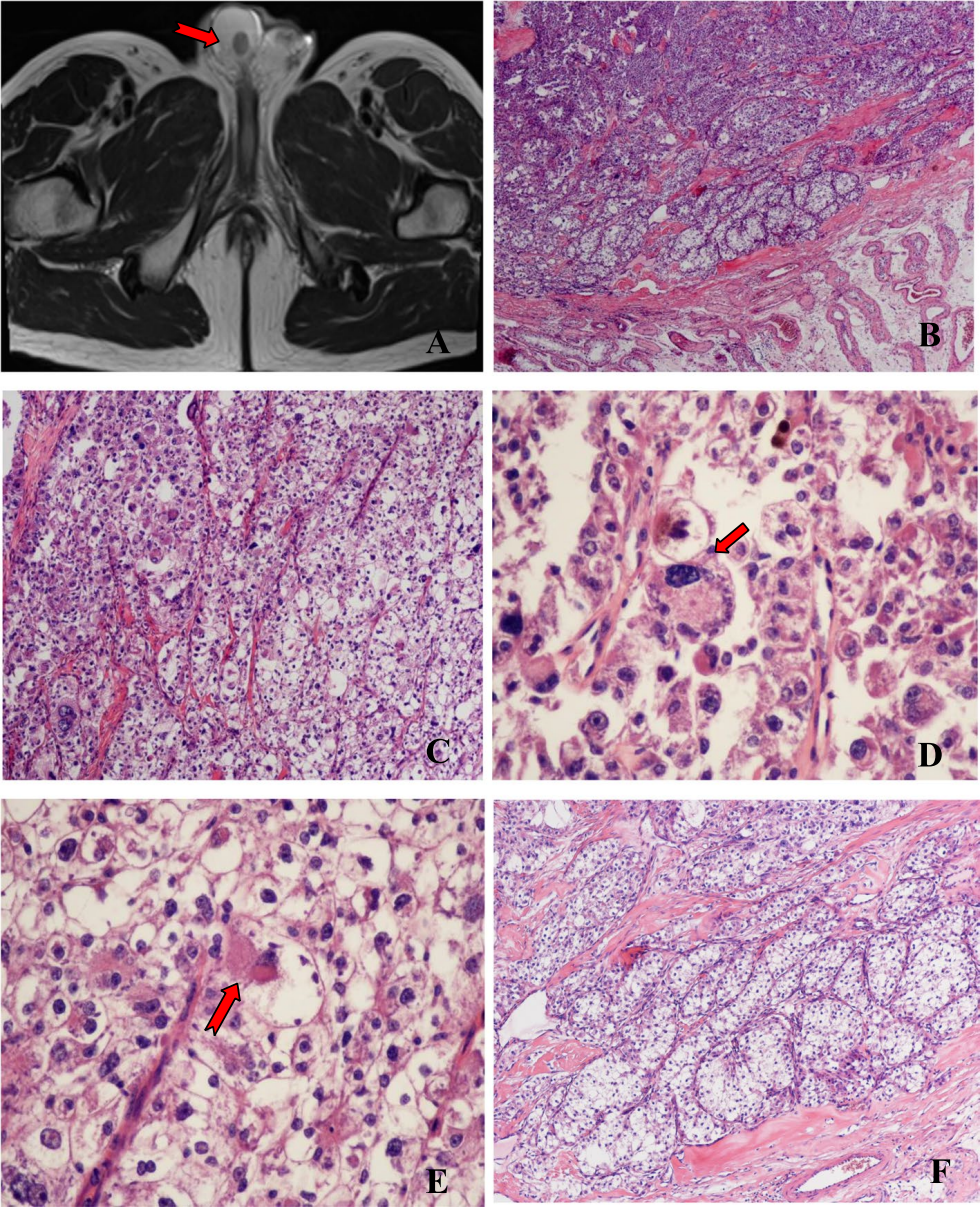
**A** MRI revealed a mass in the right testis (red arrow). **B** The tumor showed limited infiltration into the surrounding testicular tissue and displayed nested or sheet-like architecture separated by delicate fibrovascular septa. **C** Epithelioid tumor cells exhibited marked nuclear atypia and pleomorphism, with abundant clear to granular epsinophilic cytoplasm. **D** Multinucleate giant tumour cell with melanin pigment (red arrow). **E** Spider-like tumour cell (red arrow). **F** The tumor cells had clear-to-granular cytoplasm, distinct cell borders and round or oval nuclei with small visible nucleoli

A 57-year-old male touched a mass in his testis incidentally by himself a month ago, without pain, redness and swelling or urinary irritation symptoms. There was no evidence of family history, tuberous sclerosis complex (TSC) or tumor elsewhere on comprehensive and systematic work-up. Magnetic resonance imaging (MRI) of the pelvic cavity revealed a 10 mm diameter mass in the right testis (Fig. 1A). No distant organ or lymph node metastases were detected. Radical orchiectomy was performed. Grossly, the mass was well demarcated from the surrounding testicular tissue and had a solid, yellow-tan and fleshy cut surface. Microscopically, the tumor showed limited infiltration into the surrounding testicular tissue. The tumor mainly displayed nested or sheet-like architecture separated by delicate fibrovascular septa (Fig. 1B). Many of the tumor cells exhibited marked nuclear atypia and pleomorphism (Fig. 1C), significant variation in cell size and multinucleation. Multinucleate giant cells (Fig. 1D) and spider-like cells (Fig. 1E) were readily seen, with abundant granular epsinophilic cytoplasm and prominent nucleoli. A few giant cells had coarse cytoplasmic stippling (basophilic to purple cytoplasmic granules). The tumor cells in the local region of the tumor had clear-to-granular cytoplasm, distinct cell borders and round or oval nuclei with small visible nucleoli (Fig. 1F). Thick stromal collagen bands could be seen between some neoplastic cell nests. Melanin pigment was focally identified. The mitotic count was up to 2 per 10 high-power fields, and necrosis was not found.

**Fig. 2:**
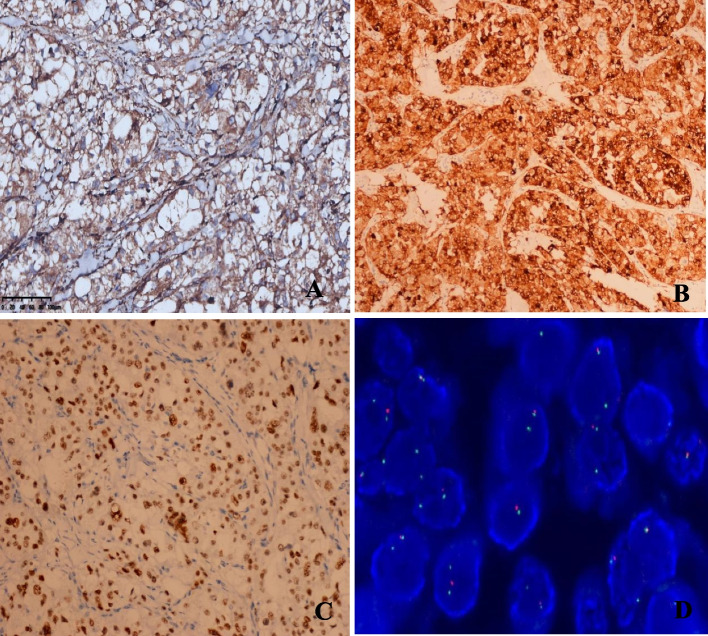
The tumor cells were diffusely strongly positive for cathepsin K (**A**) and HMB45 (**B**). (**C**) The tumor nuclei were diffusely strongly positive for TFE3. (**D**) The fusion probe assay using probes centromeric to *PSF* and telomeric to *TFE3* indicated an abnormal signal pattern consistent with the *SFPQ*-*TFE3* fusion

Immunohistochemical staining showed that the tumor cells were strongly positive for cathepsin-K (Fig. 2A), HMB45 (Fig. 2B) and TFE3 (Fig. 2C) and completely negative for PAX8, AE1/AE3, EMA, CD10, CK7, CK20, calretinin, inhibin, Melan A, S100, WT1, SMA, desmin, MyoD1, chromogranin, synaptophysin, CD56, CD68, D2-40, hepatocyte, GS and SALL4. The Ki-67 index was approximately 5%. The histomorphological findings and immunohistochemical profile were found to be consistent with Xp11 translocation PEComa.

To confirm the presence of a *TFE3* gene rearrangement, the break-apart fluorescence in situ hybridization (FISH) assay was performed on formalin-fixed, paraffin-embedded tissue sections. The case exhibited *TFE3* rearrangement with Xp11.2 translocation. The fusion probe assay using probes centromeric to *SFPQ* and telomeric to *TFE3* revealed fusion signals (Fig. 2D), thus confirming the diagnosis of Xp11 translocation PEComa.

Since the tumor was so small and the cut margin of the resected sample was tumor-free, adjuvant chemotherapy was not planned. Although the follow-up time was relatively limited, the patient was alive and well without local recurrence and distant metastasis 18 months after the surgery.

## Discussion and conclusions

The *TFE3* gene, which is located on the short arm of chromosome Xp11.2, is a member of the micropathalima-associated transcription factor family along with *TFEB*, *TFEC*, and *MiTF*. The *TFE3*-rearranged tumors mainly include Xp11 translocation renal cell carcinoma (RCC), alveolar soft part sarcoma (ASPS), PEComas/melanotic Xp11 neoplasm or Xp11 neoplasm with melanocytic differentiation, a subset of epithelioid hemangioendotheliomas and some ossifying fibromyxoid tumors [6]. Xp11 translocation PEComa reported in the literature occurred in younger patients, with a female predominance. The tumor arose in a wide range of anatomic sites, such as the kidney, ovary, pelvis, nasal cavity, adrenal gland, stomach, appendix, cervix, prostate, soft tissue, small intestine, uterus, eye and mesentery [3–5, 7–11]. The tumor sizes ranged from 1 to 17 cm (mean: 5.5 cm). Our case occurred in a relatively older male, which was the first case of a primary Xp11 translocation PEComa of the testis. The tumor size of the present case was small, with the largest dimension measuring 1.0 cm.

Because of its rarity along with nonspecific clinical presentations and imaging features, Xp11 translocation PEComa is very difficult to accurately diagnose preoperatively. It also causes diagnostic challenges to practical pathologists. Attention to its characteristic morphology, including epithelioid cells with clear or granular eosinophilic cytoplasm arranged in nested or sheet-like architectures and separated by delicate vascular networks, with the judicious use of proper immunohistochemical markers, including melanocytic markers (HMB45/MelanA), PAX8, SMA, TFE3 and cathepsin K, can help to arrive at the correct diagnosis. However, an accurate diagnosis requires molecular genetic analyses to confirm the presence of *TFE3* rearrangement. The *SFPQ*-*TFE3* gene fusion has been found to be the most common gene fusion in this rare entity. A much smaller proportion of cases have been reported with *NONO*-*TFE3* fusion [5, 12]. Rare examples demonstrated a *DVL2*, *MED15* and *RBMX* fusion partner [8–10, 13].

The main differential diagnosis of our case included Leydig cell tumor (LCT), metastatic Xp11 translocation RCC and ASPS. LCT is a neoplasm composed of cells with abundant eosinophilic cytoplasm, uniform and round tumor cells, round nuclei with prominent central nucleoli and minimal cytological atypia. Focal areas of the present tumor morphologically resembled LCT. LCT is typically positive for inhibin, calretinin, CD99 and melan A. However, nuclear TFE3 is absent. Another important differential diagnosis is with metastatic Xp11 translocation RCC, which has similar morphological features and identical gene fusion. However, Xp11 translocation PEComa is a mesenchymal tumor different from Xp11 translocation RCC. Immunophenotypic differences (absence of renal tubular markers) can distinguish the neoplasm from Xp11 translocation RCC. However, not all Xp11 translocation RCC labels renal tubular markers (including PAX8), and the clinical history is important (evidence of tumors involving kidney). ASPS shares similar clinical characteristics and morphological features with Xp11 translocation PEComa. ASPS also occurs mainly in adolescents and young adults, with a female predominance. ASPS commonly involves deep soft tissues of the extremities. ASPS is composed of epithelioid cells with eosinophilic to clear cytoplasm arranged in a uniform organoid or nest-like pattern delineated by delicate fibrovascular septa. The tumor cells show central discohesion resulting in the characteristic alveolar pattern. ASPS also shows immunopositivity for cathepsin K and nuclear immunoreactivity for TFE3. However, ASPS is negative for melanocytic markers. Cytogenetically, ASPS is defined by a specific alteration, der(17)t(X;17)(p11;q25), which results in *ASPSCR1*-*TFE3* gene fusion. *ASPSCR1*–*TFE3* translocation is known to be the characteristic diagnostic gene fusion for ASPS among sarcomas.

Xp11 translocation PEComa has special clinicopathological features compared to conventional PEComas, such as alveolar/nested growth pattern of large epithelioid cells with clear to eosinophilic cytoplasm, lack of spindle cell or fat components, negative for SMA, *TFE3* gene rearrangement and absence of *TSC1*/*2* genetic alterations. Xp11 translocation PEComa had a more aggressive behavior. In the study by Rao et al., they summarized 24 cases of this unique entity in the literature: 15 of 24 (62.5%) cases experienced recurrence and/or metastases or died of disease [9]. A recent study on the largest series of melanotic Xp11 neoplasms showed local recurrence and distant metastatic rates of 31.8% and 31.8%, respectively [10]. Xp11 translocation PEComa with a novel RBMX-TFE3 gene fusion in a pediatric kidney presented with a fulminant clinical course (multiple pulmonary metastases at diagnosis and died of disease 3 months later) [13]. While optimal treatment for Xp11 translocation PEComa has not been established, surgical resection appears to be the mainstay of the therapy. PEComa patients with *TSC* mutations with locally aggressive or metastatic disease can use palliative therapy with mTOR inhibitors [14]. Recently, Schmiester et al. [15] reported a clinically aggressive *TSC1*-mutated PEComa with concomitant *TFE3* activation. It is unclear whether *TFE3*-associated PEComa patients can respond to mTOR inhibitors. *TFE3* transcription triggers Met receptor tyrosine kinase by direct transcriptional upregulation, leading to the activation of downstream pathways, such as the PI3K/AKT/mTOR pathway. It is possible that patients with Xp11 translocation PEComa might benefit from tyrosine kinase inhibitors.

In conclusion, we described the first case of a primary Xp11 translocation PEComa arising within the testis in a relatively older man harboring the *SFPQ*-*TFE3* gene fusion by FISH. It is difficult to predict the prognosis, and a treatment strategy has not been established. The accumulation of further cases and long-term follow-up data are desirable.

## Data Availability

Not applicable.

## Abbreviations

PEComa: Perivascular epithelioid cell tumor
RCC: Renal cell carcinoma
ASPS: Alveolar soft part sarcoma
LCT: Leydig cell tumor
